# Alpha Smooth Muscle Actin (αSMA) Immunohistochemistry Use in the Differentiation of Pancreatic Cancer from Chronic Pancreatitis

**DOI:** 10.3390/jcm10245804

**Published:** 2021-12-11

**Authors:** Katarzyna Winter, Monika Dzieniecka, Janusz Strzelczyk, Małgorzata Wągrowska-Danilewicz, Marian Danilewicz, Ewa Małecka-Wojciesko

**Affiliations:** 1 Clinical Department of General and Oncological Gastroenterology, University Clinical Hospital No. 1, 90-153 Lodz, Poland; ewuncia@poczta.onet.pl; 2 Sykehuset Innlandet HF, 2618 Lillehammer, Norway; m.dzienieck2@gmail.com; 3 Department of General and Transplant Surgery, Medical University of Lodz, 90-153 Lodz, Poland; janusz.strzelczyk@umed.lodz.pl; 4 Department of Nephropathology, Division of Morphometry, Medical University of Lodz, 90-153 Lodz, Poland; malgorzata.wagrowska-danilewicz@umed.lodz.pl (M.W.-D.); marian.danilewicz@umed.lodz.pl (M.D.); 5 Department of Digestive Tract Diseases, Medical University of Lodz, 90-153 Lodz, Poland

**Keywords:** chronic pancreatitis, pancreatic ductal adenocarcinoma, pancreatic fibrosis, pancreatic stellate cells, αSMA

## Abstract

Aim: Fibrosis is observed both in pancreatic cancer (PDAC) and chronic pancreatitis (CP). The main cells involved in fibrosis are pancreatic stellate cells (PSCs), which activate alpha smooth muscle actin (αSMA), which is considered to be the best-known fibrosis marker. The aim of the study was to evaluate the expression of the αSMA in patients with PDAC and CP as the possible differentiation marker. Methods: We enrolled 114 patients undergoing pancreatic resection: 83 with PDAC and 31 with CP. Normal fragments of resected specimen from 21 patients represented the control tissue. The immunoexpressions of αSMA were detected in tissue specimens with immunohistochemistry (Abcam antibodies, GB). Results: Mean cytoplasmatic expression of αSMA protein in PDAC stromal cells was significantly higher compared to CP: 2.42 ± 0.37 vs 1.95 ± 0.45 (*p* < 0.01) and control group 0.61 ± 0.45 (*p* < 0.01). Strong immunoexpression of the αSMA protein was found in the vast majority (80.7%) of patients with PDAC, in about half (58%) of patients with CP, and not at all in healthy tissue. The expression of αSMA of different intensity was found in all patients with PDAC and CP, while in healthy tissue was minimal or absent. In PDAC patients, αSMA expression was significantly higher in tumors of diameter higher than 3 cm compared to smaller ones (*p* = 0.017). Conclusions: Presented findings confirm the significant role of fibrosis in both PDAC and CP; however, they do not confirm the role of αSMA as a marker of differentiation.

## 1. Introduction

Chronic pancreatitis is a long-term inflammatory process that leads to irreversible morphological changes in the pancreatic parenchyma, its gradual fibrosis, and calcification [1,2,3,4]. A similarly characteristic feature of the pancreatic cancer is the fibrous stroma, which is the main ingredient of the tumor [5,6,7,8]. Long-term CP is an important risk factor for the development of pancreatic cancer, although the mechanisms leading to cancer transformation are still poorly understood [9,10]. It is believed that chronic inflammatory process underlies malignant transformation [9,10,11,12]. Pancreatic chronic inflammatory process leads to the activation of stellate cells. Activated stellate cells can proliferate and migrate, are a source of extracellular matrix (ECM) proteins, pro-inflammatory cytokines (IL-1, IL-6, IL-8, IL-18, IL-33, TNFα), chemokines and growth factors (incl. IGF-1, PDGF, TGF-β1) [6,7,8]. As a result of activation, stellate cells acquire a muscle cell phenotype, which express alpha smooth muscle actin (αSMA), considered the best-known fibrosis marker [6,7,8]. Fibrous stroma of the pancreatic tumor represents the source of numerous factors responsible for the extraordinary aggressiveness of this tumor, plays an important role in the progression and metastasis formation as well as prevents the access of chemotherapeutics to the cancer cells [13,14,15,16,17].

The differentiation of focal lesions arising from chronic pancreatitis and PDAC remains a diagnostic challenge [18]. At the present time, endoscopic ultrasound (EUS) is a well-established tool for the evaluation of pancreatic lesions, which offers a high sensitivity for detection of small pancreatic mass and is the preferred modality for obtaining tissue for diagnosis of pancreatic mass [19]. Fine-needle biopsy (FNB) was recently developed to obtain a core tissue, providing samples with preserved architecture for histological, immunohistochemical profiling [20,21]. Immunohistochemistry is an integral technique for tissue-based diagnostics and biomarker detection with broad worldwide adoption. Advances in core chemistries, antibody design, and automation have ushered unprecedented sensitivity, specificity, and reproducibility in immunohistochemistry assays [22].

Numerous studies have been conducted for many years, focusing on the search for a specific, sensitive diagnostic and prognostic marker for pancreatic cancer, but their results are still unsatisfactory. The aim of the study was to evaluate the expression of the αSMA in patients with PDAC and CP as the possible differentiation marker.

## 2. Material and Methods

The study included 114 patients hospitalized and diagnosed in the Department of Gastrointestinal Diseases at the Medical University of Lodz and then operated at the Department of General and Transplant Surgery at the Medical University of Lodz—83 patients with ductal pancreatic adenocarcinoma and 31 patients with chronic pancreatitis. The control represents the fragments of normal pancreas in preparations obtained from 21 patients from the study group.

Initial diagnosis was based on the clinical evaluation and additional biochemical and imaging tests (abdominal ultrasound, CT scans, magnetic resonance, EUS). The general indication for surgery was the presence of a focal lesion in the pancreas. In the case of pancreatic cancer, the resectability criteria included: no distant metastases and no signs of infiltration of the superior mesenteric vein and/or portal vein in imaging or infiltration enabling safe resection and vascular reconstruction [23]. The final diagnosis of the disease was based on the histopathological examination of the postoperative material.

In patients with PDAC, tumor size, location, histological grade and tumor stage according TNM classification were assessed [24]. Survival was determined in days, calculated from the date of surgery to the date of death.

For immunohistochemistry anti actin (Smooth Muscle) (Dako nr kat.M0851) was used. The αSMA expression has been reported in the cytoplasm of pancreatic stromal cells.

The degree of immunoexpression of αSMA was determined based on the intensity of the color and the number of stained cells. The degree of immunoreactivity was assessed by semi-quantitative method assigning from 0 to 3 points depending on the intensity of the reaction: 0 in the absence of an immunohistochemical reaction, 1 in the case of a weak, 2 moderate and 3 a strong immunohistochemical reaction. The percentage of stained cells was also assessed on a three-point scale, assigning 1: for less than 30%, 2: for 31 to 65% and a value of 3 when more than 66% of the cells were stained. In each of the preparations, ten fields of view were evaluated at 400× magnification and the average score calculated for each sample. Then, the average value was calculated from both obtained values and subjected to statistical analysis.

The following tests were used during the statistical analysis of the collected data: to check the normal distribution Shapiro–Wilk test; for comparisons of measurable features between groups Mann–Whitney U test; for all comparisons of immeasurable features test of independence. For all statistical tests used, the level of statistical significance was assumed at *p* < 0.05. Statistical analysis was performed using the Statistica 12 program.

The study was approved by the Bioethics Committee of the Medical University of Lodz (approval No. RNN/202/12/EC of 20 November 2012).

## 3. Results

In the group with PDAC were 46 men and 37 women, aged 34–76 (mean age: 59.6 ± 8.74 years). Among patients with chronic pancreatitis, there were 22 men and 9 women, aged 23–64 (mean age: 49.7 ± 9.65 years). When analyzing the clinical symptoms, jaundice was significantly more frequent in patients with PDAC (*p* < 0.02) than in CP patients. Additionally, mean fasting glucose (*p* = 0.05) and BMI index (*p* < 0.05) were statistically significantly higher in PDAC patients compared to patients with CP. There was no statistically significant difference between PDAC and CP regarding the mean serum CA 19-9 level (*p* = 0.1383). Based on imaging tests, intraoperative assessment and histopathological examination results, PDAC staging was assessed according to TNM classification. The baseline characteristic of the subjects involved in the study are presented in Table 1.

The average survival time for patients with PDAC was 1.4 years. Seven pancreatic cancer patients (8.4%) survived until the end of follow-up. The average survival time in these 7 PDAC patients was 5.24 years (min. 3.3 years, max. 7.6 years). In the group of CP patients, 10 people (32%) died by the end of follow-up. The maximum follow-up period was 10.17 years.

**Figure 1:**
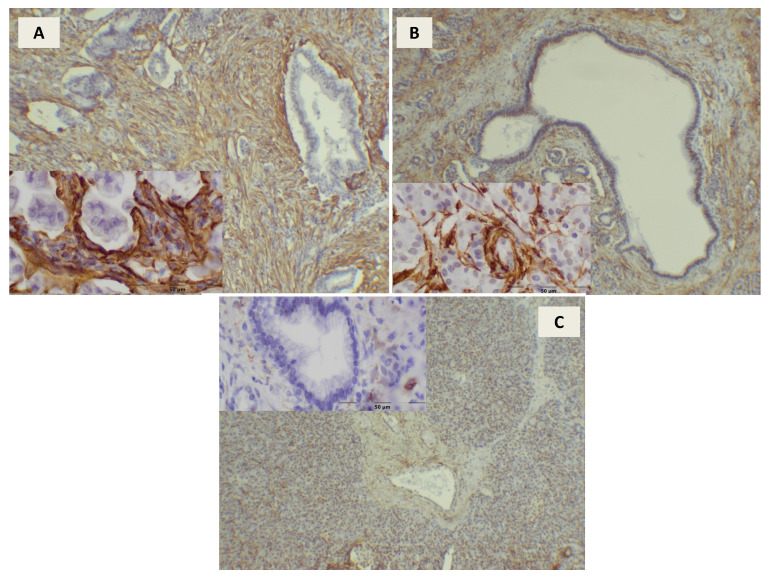
Immunoexpression of αSMA in pancreatic ductal adenocarcinoma - intensity of the reaction 3 (**A**), chronic pancreatitis - intensity of the reaction 2 (**B**) and in the control group - intensity of the reaction 0 (**C**) 100 and 400× magnification.

**Figure 2:**
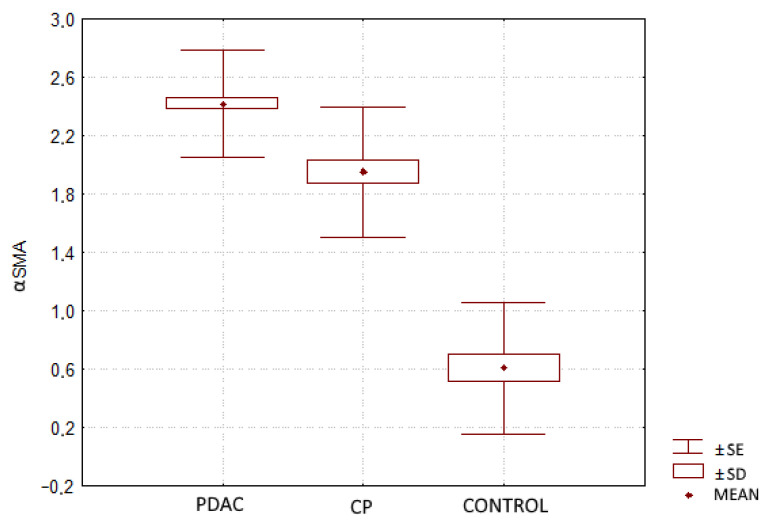
Comparison of αSMA expression in pancreatic ductal adenocarcinoma (PDAC), chronic pancreatitis (CP) and in healthy tissue (CONTROL).

Immunohistochemical analysis showed cytoplasmic expression of the αSMA protein in pancreatic stromal cells (Figure 1). Significantly higher mean expression of the αSMA protein in PDAC was demonstrated in comparison to CP: 2.42 ± 0.37 vs. 1.95 ± 0.45 (*p* < 0.01). The expression of αSMA in the control group was 0.61 ± 0.45, which was 4 times lower than in PDAC and 3 times lower than in CP. The differences between all groups were statistically significant (*p* < 0.01) (Figure 2).

**Figure 3:**
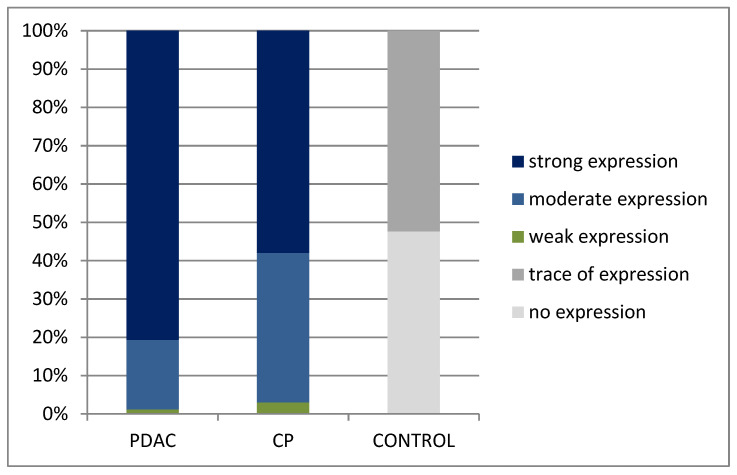
Comparison of intensity of αSMA immunoexpression in pancreatic ductal adenocarcinoma (PDAC), chronic pancreatitis (CP) and in healthy tissue (CONTROL).

Strong immunoexpression of the αSMA protein was found in 67 (80.7%) of pancreatic cancer patients and in 16 (58%) of CP patients, and not at all in healthy tissue. In patients with PDAC moderate expression was revealed in 15 patients (18.1%) and weak in 1 patient (1.2%). αSMA of different intensity was expressed in all patients with PDAC and CP. In healthy tissue, in all cases, this expression was only residual (11 patients 52.4%) or absent (10 patients 47.6%). It may be suggested that moderate or strong αSMA expression clearly differentiates CP and PDAC from healthy tissue. The results are shown in Figure 3.

In the studied group, we have found no correlation between αSMA immonoexpression and age, sex, jaundice, serum CA19-9 level, diabetes mellitus, including newly diagnosed, and tumor location.

**Figure 4:**
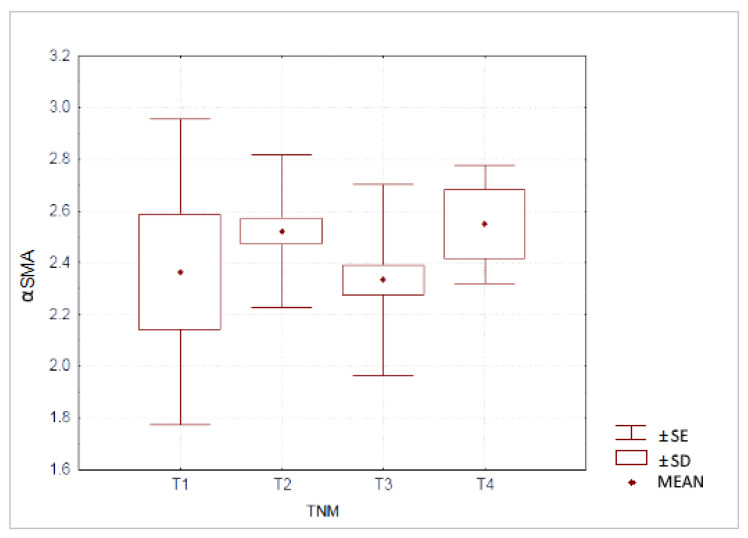
Expression of αSMA in pancreatic ductal adenocarcinoma according to TNM classification.

We also analyzed relationship between fibrosis and the advancement of the neoplastic process according to TNM classification. The mean expression of αSMA was in: T1 2.36 ± 0.59, T2 2.52 ± 0.29, T3 2.33 ± 0.37 and in T4 2.55 ± 0.23. The differences in αSMA expression between those subgroups were not statistically significant and are shown in Figure 4.

**Figure 5:**
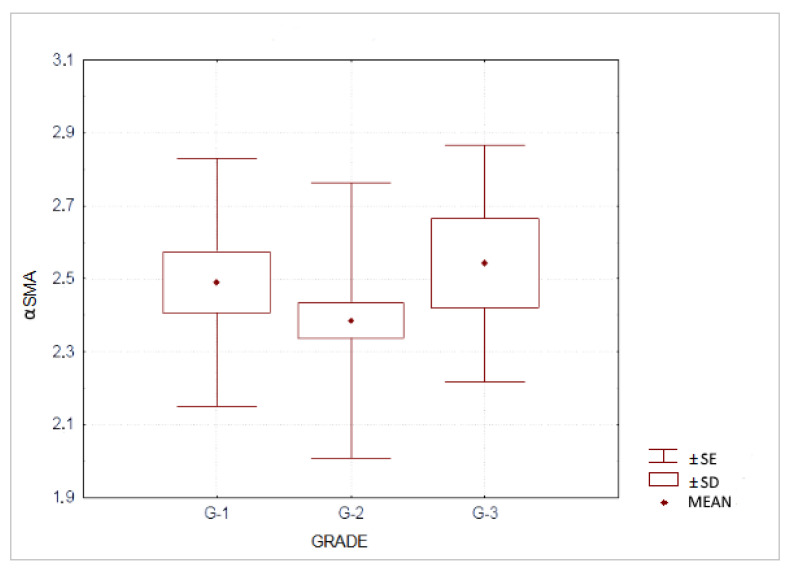
Expression of αSMA in pancreatic ductal adenocarcinoma according to the degree of histological differentiation.

Furthermore, we analyzed αSMA expression according to the degree of histological differentiation. We revealed the highest expression of αSMA protein in G-3 tumors 2.54 ± 0.32 vs G-1 tumors 2.49 ± 0.34 and G-2 tumors 2.39 ± 0.38. However, the differences between all those subgroups were not statistically significant (G-1 vs. G-2 *p* = 0.2181; G-1 vs. G-3 *p* = 0.9196 and G-2 vs. G-3 *p* = 0.3245). Results are shown in Figure 5.

**Figure 6:**
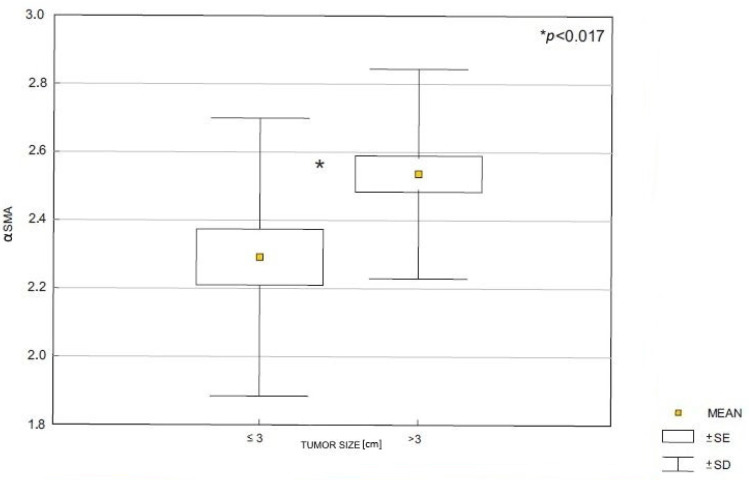
Comparison of αSMA expression in tumors > and ≤ 3 cm.

Significantly higher expression of αSMA was found in tumors above 3 cm 2.54 ± 0.31 compared to tumors ≤3cm 2.29 ± 0.40 (*p* = 0.0177) Figure 6.

To assess the possibility of differentiating PDAC and CP based on the determined αSMA expression, ROC curves and graphs of sensitivity and specificity were prepared. At cut-off point 2.45 the sensitivity was 30.1%, specificity 35.5%, PPV 55.6%, NPV 15.9%.

**Figure 7:**
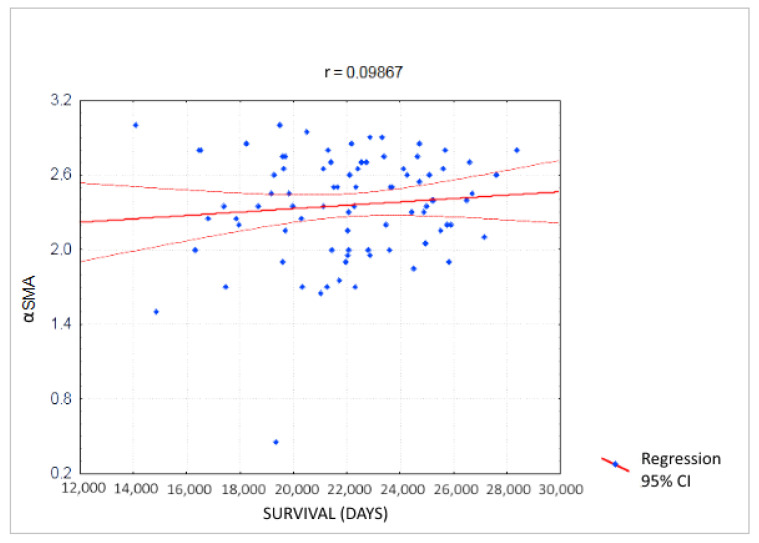
Correlation between the expression of the αSMA protein and the survival time in pancreatic ductal adenocarcinoma patients.

We did not find any correlation between the expression of the αSMA protein and the survival time of PDAC patients (r = 0.09867) (Figure 7).

## 4. Discussion

To assess fibrosis in the analyzed group of patients, we assessed the immunoexpression of αSMA protein, which is considered to be one of the best-known fibrosis marker. We confirmed the expression of αSMA in the cytoplasm of pancreatic stromal cells in both cancer and CP. In PDAC patients, this expression was significantly higher than in patients with CP. Strong expression of αSMA protein was seen in vast majority (81%) of PDAC patients and in about half (58%) of CP patients. In turn, in normal pancreas, the expression of αSMA was significantly lower compared to PDAC patients and was residual or absent. Similar results were obtained by Cyriac et al., in human postoperative specimen, where significantly higher immunoexpression of the αSMA protein in PDAC compared to the alcoholic CP (*p* = 0.02) and normal pancreas (*p* < 0.001) was found [25]. They also showed a significantly lower expression of the αSMA protein in the normal pancreas than in alcohol-related CP (*p* = 0.003). On the other hand, Sinn et al., in 162 patients after radical pancreatic cancer surgery, found strong αSMA protein expression in 46 (29%), moderate in 87 (54%) and weak in 25 (16%) patients [26]. Similar results were obtained by Wang et al., in 142 PDAC patients after radical surgery [27]. They confirmed the strong and moderate αSMA expression in 36 patients (24.88%) and 77 patients (53.1%), respectively, and low or none in 32 patients (22.1%). These results confirm a significant activation of fibrosis in both CP and PDAC. Data from our study as well from the others suggest that strong and moderate αSMA expression clearly differentiates CP and PDAC from healthy tissue.

To assess the possibility of differentiating PDAC and CP based on αSMA expression, ROC curves and graphs of sensitivity and specificity were prepared. At cut-off point of 2.45, the sensitivity was 30.1%, specificity 35.5%, PPV 55.6%, NPV 15.9%. These results indicate that αSMA is not suitable for the differential diagnosis between PDAC and CP.

Analyzing the difference between fibrosis in PDAC and CP, Drifka et al., confirmed in human material, the unique collagen topology in the periductal stroma of pancreatic ductal adenocarcinoma [28]. It was found that the collagen fibers around cancer cells are longer and wider compared to the normal pancreas and CP. Perhaps further examination of the collagen fibers may help in more detailed differentiation of benign and malignant pancreatic tumors. In our study, as well as in the others, PDAC was characterized with a higher degree of fibrosis, expressed as the degree of αSMA expression, compared to CP and normal tissue. We assume that EUS-FNB histological biopsy with αSMA evaluation in the obtained material in addition to EUS elastography may enhance the possibilities of PDAC and CP differentiation.

We did not find a relationship between the expression of the αSMA protein and the PDAC stage, differentiation degree and patients’ survival. However, we showed the highest αSMA protein expression in poorly differentiated tumors, suggesting greater fibrosis in more aggressive tumors. Similarly, Sinn et al., analyzing the expression of αSMA in 162 patients with resectable PDAC, showed no correlation between αSMA expression and the cancer stage in the TNM classification, the degree of histological differentiation, lymph node metastases and R0 resection [26]. However, Erkan et al., showed that the combination of high αSMA and low collagen expression, defined as an activated stromal index, was associated with a worse prognosis, but expression of αSMA itself did not have the prognostic significance [29]. Wang et al., found in a multivariate analysis that the high density of fibrous tissue assessed by H&E staining in PDAC patients is an independent prognostic factor for shorter overall survival (*p* = 0.001), shorter progression-free survival (*p* = 0.007) shorter local progression-free survival (*p* = 0.001) and shorter distant metastatic free survival (*p* = 0.002) [27]. On the other hand, in the multivariate analysis, the researchers did not confirm the prognostic significance of αSMA protein immunoexpression in PDAC. They also found a significant positive correlation between low αSMA expression and G1 tumor differentiation and negative with G2–G3 differentiation. Those data indicate that although the role of αSMA in PDAC-associated fibrosis is recognized, its role in the tumor progression is not clearly elucidated yet and needs further studies.

In our study, we confirmed the relationship between αSMA expression and the size of the pancreatic tumor. We found significantly higher expression of αSMA in tumors over 3 cm compared to tumors <3 cm (*p* = 0.0177). It could be assumed that locally advanced tumors are characterized by a greater amount of fibrous tissue, which affects their aggressiveness and worse prognosis. In a study by Hwang et al., stellate cells were isolated from postoperative pancreatic cancer material, identified with immunohistochemistry e.g., for αSMA and injected into the mouse PDAC model [16]. The administration of stellate cells increased the tumor size and the ability to metastasize, which confirms the important role of fibrosis in pancreatic carcinogenesis as well as tumor progression. Wantanabe et al., analyzing the postoperative PDAC material confirmed that increased fibrosis was associated with a shorter survival [30]. Similarly, Fujita et al., analyzing the expression of αSMA RNA in 109 patients after pancreatic cancer surgery, confirmed a significant correlation between high αSMA expression and shorter survival time, the association with increased invasiveness and tumor cell proliferation [31]. In a randomized phase III trial with the use of gemcitabine as an adjuvant treatment in resected PDAC, it was confirmed that high αSMA expression was associated with shorter disease-free time and survival [26]. In a study by Marechal et al., a high stromal index, expressed as the amount of stromal tissue in relation to neoplastic cells was also associated with a worse prognosis [32]. Those data correspond with our findings on high αSMA expression in large tumors and indicate on the possible prognostic role of this protein in PDAC.

The relationship between the degree of fibrosis in pancreatic tumors and the prognosis suggests the possibility for the elaborating the treatment aiming at fibrosis inhibition, allowing the penetration of chemotherapeutic agents into the cancer cells, thus providing a chance for longer survival of the PDAC patients. Froeling et al., by inducing the dormant state of stellate cells with trans-retinoic acid, achieved the reduction in cancer cell proliferation, induction of apoptosis, as well tumor growth and invasiveness reduction in a transgenic PDAC mouse model [33]. In a study by Alvarez et al., the use of nab-paclitaxel and gemcitabine in advanced PDAC reduced the content of tumor-associated fibroblasts [34]. In another study, performed in mouse PDAC models, inhibition of the stromal reaction in tumor, by adding nab-paclitaxel to gemcitabine increased intra-tumor concentrations of gemcitabine 2.8-fold, compared to gemicitabine alone [35]. In a study by Miyashita et al., the effects of neoadjuvant chemotherapy on cancer-associated fibroblasts (CAFs) in pancreatic cancer stroma were investigated. αSMA expression was reduced in the gemcitabine plus nab-paclitaxel group, as revealed by markedly disorganized collagen and a low density of αSMA-positive fibroblasts [36]. The effects of nab-paclitaxel on PDAC stroma are not fully understood. It is suggested that they include stromal modelling with marked changes in collagen architecture and elimination of CAFs [34].

Immunohistochemistry is an integral technique for tissue-based diagnostics and biomarker detection that has been widely adopted around the world. Advances in core chemistries, antibody design, and automation have resulted in unprecedented sensitivity, specificity, and reproducibility in immunohistochemistry assays [22,37]. Clinical immunohistochemistry assays that use mutation-specific antibodies provide novel tools in clinical diagnostics. Multiplex and mutation-specific immunohistochemistry assays represent important innovations that provide improved utility in the context of personalized medicine and targeted therapy [23].

For the time being, our findings have very limited practical clinical application. However, EUS-guided fine-needle biopsy (EUS-FNB) is the best way for obtaining a histological material, which may be used for immunohistochemistry [38,39]. Fibrosis assessment using less invasive methods, i.e., EUS-FNB with αSMA immunohistochemistry, could identify patients, who would be more resistant to chemotherapy due to greater stromal fibrosis, and would require fibrosis-inhibiting treatment in addition to standard chemiotherapy. These data may be also helpful in elaborating future cancer treatments timing at specific molecules.

## 5. Conclusions

Presented findings support the data of the significant role of fibrosis in PDAC and CP and confirm that the moderate or high expression of αSMA differentiates CP and PDAC from healthy tissue. However, they do not confirm the role of αSMA as a marker of pancreatic cancer differentiation from chronic pancreatitis. Since αSMA is expressed in almost all PDAC specimen and not in the healthy tissue it may be probably used as an additional tool, together with other biomarkers in PDAC diagnosis.

## Figures and Tables

**Table 1:** Baseline Characteristics of the Study Group.

Baseline Characteristics of the Study Group			
Number of Patients		PDAC Patients	CP Patients
		83	31
Age		59.6 ± 8.74 (34–76)	49.7 ± 9.65 (23–64)
Sex	Female	37 (45%)	9 (29%)
	Male	46 (55%)	22 (71%)
Symptoms	Jaundice	37 (45%)	5 (16%)
BMI		25.15 ± 3.73	22.84 ± 4.72
Blood tests	Fasting glucose	118.44 ± 34.02	103.26 ± 20.05
	CA 19-9 serum level	217.3 ± 348.7	68.3 ± 135.8
Comorbidities	DM	29 (47%)	11 (35.5%)
	DM newly diagnosed	12 (14.5%)	3 (9.6%)
TNM Classification	IA	5 (6%)	n/a
	IB	21 (25%)	n/a
	IIA	19 (23%)	n/a
	IIB	32 (38.5%)	n/a
	III	3 (3.6%)	n/a
	IV	3 (3.6%)	n/a
Tumor Grading	G-1	16 (19.3%)	n/a
	G-2	60 (72.3%)	n/a
	G-3	7 (8.4%)	n/a
Localization	Head of the pancreas	68 (82%)	27 (87%)
	Body of the pancreas	8 (9.6%)	0
	Tail of the pancreas	7 (8.4%)	4 (13%)
Tumor Dimension [cm]		3.45 (0.5–6.0)	n/a
Type of operations	Whipple’s operation	63 (76%)	25 (81%)
	Resection of the body and tail of pancreas	8 (9.6%)	0
	Resection of the tail of the pancreas	3 (3.6%)	4 (13%)
	Beger operation	0	2 (6%)
	Total pancreatectomy	9 (10.8%)	0

PDAC—pancreatic ductal adenocarcinoma; CP—chronic pancreatitis; BMI—body mass index; DM—diabetes mellitus; n/a—not applicable.

## References

[1] Braganza J.M., Lee S.H., McCloy R.F., McMahon M.J. (2011). Chronic pancreatitis. Lancet.

[2] Witt H., Apte M.V., Keim V., Wilson J.S. (2007). Chronic pancreatitis: Challenges and advances in pathogenesis, genetics, diagnosis, and therapy. Gastroenterology.

[3] Etrnad B., Whitcomb D.C. (2001). Chronic pancreatitis: Diagnosis, classification, and new genetic developments. Gastroenterology.

[4] Manohar M., Verma A.K., Venkateshaiah S.U., Sanders N.L., Mishra A. (2017). Pathogenic mechanisms of pancreatitis. World J. Gastrointest. Pharmacol. Ther..

[5] Apte M.V., Park S., Phillips P.A., Santucci N., Goldstein D., Kumar R.K., Ramm G.A., Buchler M., Friess H., McCarroll J.A. (2004). Desmoplastic reaction in pancreatic cancer: Role of pancreatic stellate cells. Pancreas.

[6] Apte M.V., Pirola R.C., Wilson J.S. (2012). Pancreatic stellate cells: A starring role in normal and diseased pancreas. Front. Physiol..

[7] Pandol S., Edderkaoui M., Gukovsky I., Lugea A., Gukovskaya A. (2009). Desmoplasia of pancreatic ductal adenocarcinoma. Clin. Gastroenterol. Hepatol..

[8] Bachem M.G., Zhou S., Buck K., Schneiderhan W., Siech M. (2008). Pancreatic stellate cells—Role in pancreas cancer. Langenbecks Arch. Surg..

[9] Raimondi S., Lowenfels A.B., Morselli-Labate A.M., Maisonneuve P., Pezzilli R. (2010). Pancreatic cancer in chronic pancreatitis; aetiology, incidence, and early detection. Best Pract. Res. Clin. Gastroenterol..

[10] Lowenfels A.B., Maisonneuve P., Cavallini G. (1993). Pancreatitis and the risk of pancreatic cancer. N. Engl. J. Med..

[11] Farrow B., Sugiyama Y., Chen A., Uffort E., Nealon W., Mark Evers B. (2004). Inflammatory mechanisms contributing to pancreatic cancer development. Ann. Surg..

[12] Garcea G., Dennison A.R., Steward W.P., Berry D.P. (2005). Role of inflammation in pancreatic carcinogenesis and the implications for future therapy. Pancreatology.

[13] Vonlaufen A., Joshi S., Qu C., Phillips P.A., Xu Z., Parker N.R., Toi C.S., Pirola R.C., Wilson J.S., Goldstein D. (2008). Pancreatic stellate cells: Partners in crime with pancreatic cancer cells. Cancer Res..

[14] Xu Z., Vonlaufen A., Phillips P.A., Fiala-Beer E., Zhang X., Yang L., Biankin A.V., Goldstein D., Pirola R.C., Wilson J.S. (2010). Role of pancreatic stellate cells in pancreatic cancer metastasis. Am. J. Pathol..

[15] Dunѐr S., Lindman J.L., Ansari D., Gundewar C., Andersson R. (2010). Pancreatic cancer: The role of pancreatic stellate cells in tumor progression. Pancreatology.

[16] Hwang R.F., Moore T., Arumugam Ramachandran V., Amos K.D., Rivera A., Ji B., Evans D.B., Logsdon C.D. (2008). Cancer-associated stromal fibroblasts promote pancreatic tumor progression. Cancer Res..

[17] Heinemann V., Reni M., Ychou M., Richel D.J., Macarulla T., Ducreux M. (2014). Tumour-stroma interactions in pancreatic ductal adenocarcinoma: Rationale and current evidence for new therapeutic strategies. Cancer Treat. Rev..

[18] Wolske K.M., Ponnatapura J., Kolokythas O., Burke L.M.B., Tappouni R., Lalwani N. (2019). Chronic pancreatitis or pancreatic tumor? A problem-solving approach. Radiographics.

[19] Chong C.C.N., Tang R.S.Y., Wong J.C.T., Chan A.W.H., Teoh A.Y.B. (2016). Endoscopic ultrasound of pancreatic lesions. J. Vis. Surg..

[20] Sugimoto M., Irie H., Takagi T., Suzuki R., Konno N., Asama H., Sato Y., Nakamura J., Takasumi M., Hashimoto M. (2020). Efficacy of EUS-guided FNB using a Franseen needle for tissue acquisition and microsatellite instability evaluation in unresectable pancreatic lesions. BMC Cancer.

[21] Tiriac H., Bucobo J.C., Tzimas D., Grewel S., Lacomb J.F., Rowehl L.M., Nagula S., Wu M., Kim J., Sasson A. (2018). Successful creation of pancreatic cancer organoids by means of EUS-guided fine-needle biopsy sampling for personalized cancer treatment. Gastrointest. Endosc..

[22] Sukswai N., Khoury J.D. (2019). Immunohistochemistry innovations for diagnosis and tissue-based biomarker detection. Curr. Hematol. Malig. Rep..

[23] National Comprehensive Cancer Network NCCN Guidelines Version 1.2020 Pancreatic Adenocarcinoma. http://www.nccn.org.

[24] Amin M.B., Edge S.B., Greene F., Byrd D.R., Brookland R.K., Washington M.K., Gershenwald J.E., Compton C.C., Hess K.R., Sullivan D.C. (2017). AJCC Cancer Staging Manual.

[25] Cyriac J., Mahadevan P., Augustine P., Ramesh H., Koshy A. (2012). Stellate cell activation in tropical calcific pancreatitis compared to alcoholic pancreatitis, adenocarcinoma of pancreas and normal pancreas. JOP.

[26] Sinn M., Denkert C., Striefler J.K., Pelzer U., Stieler J.M., Bahra M., Lohneis P., Dörken B., Oettle H., Riess H. (2014). α-Smooth muscle actin expression and desmoplastic stromal reaction in pancreatic cancer: Results from the CONKO-001 study. Br. J. Cancer.

[27] Wang L.M., Silva M.A., D’Costa Z., Bockelmann R., Soonawalla Z., Liu S., O’Neill E., Mukherjee S., McKenna W.G., Muschel R. (2016). The prognostic role of desmoplastic stroma in pancreatic ductal adenocarcinoma. Oncotarget.

[28] Drifka C.R., Tod J., Loeffler A.G., Liu Y., Thomas G.J., Eliceiri K.W., Kao W.J. (2015). Periductal stromal collagen topology of pancreatic ductal adenocarcinoma differs from that of normal and chronic pancreatitis. Mod. Pathol..

[29] Erkan M., Michalski C.W., Rieder S., Reiser-Erkan C., Abiatari I., Kolb A., Giese N.A., Esposito I., Friess H., Kleeff J. (2008). The activated stroma index is a novel and independent prognostic marker in pancreatic ductal adenocarcinoma. Clin. Gastroenterol. Hepatol..

[30] Watanabe I., Hasebe T., Sasaki S., Konishi M., Inoue K., Nakagohri T., Oda T., Mukai K., Kinoshita T. (2003). Advanced pancreatic ductal cancer: Fibrotic focus and beta-catenin expression correlate with outcome. Pancreas.

[31] Fujita H., Ohuchida K., Mizumoto K., Nakata K., Yu J., Kayashima T., Cui L., Manabe T., Ohtsuka T., Tanaka M. (2010). α-Smooth muscle actin expressing stroma promotes an aggressive tumor biology in pancreatic ductal adenocarcinoma. Pancreas.

[32] Maréchal R., Bachet J.B., Calomme A., Demetter P., Delpero J.R., Svrcek M., Cros J., Bardier-Dupas A., Puleo F., Monges G. (2015). Sonic hedgehog and Gli1 expression predict outcome in resected pancreatic adenocarcinoma. Clin. Cancer Res..

[33] Froeling F.E., Feig C., Chelala C., Dobson R., Mein C.E., Tuveson D.A., Clevers H., Hart I.R., Kocher H.M. (2011). Retinoic acid-induced pancreatic stellate cell quiescence reduces paracrine Wnt-beta-catenin signaling to slow tumor progression. Gastroenterology.

[34] Alvarez R., Musteanu M., Garcia-Garcia E., Lopez-Casas P.P., Megias D., Guerra C., Muñoz M., Quijano Y., Cubillo A., Rodriguez-Pascual J. (2013). Stromal disrupting effects of nab-paclitaxel in pancreatic cancer. Br. J. Cancer.

[35] Von Hoff D.D., Ramanathan R.K., Borad M.J., Laheru D.A., Smith L.S., Wood T.E., Muñoz M., Quijano Y., Cubillo A., Rodriguez-Pascual J. (2011). Gemcitabine plus nab-paclitaxel is an active regimen in patients with advanced pancreatic cancer: A phase I/II trial. J. Clin. Oncol..

[36] Miyashita T., Tajima H., Makino I., Okazaki M., Yamaguchi T., Ohbatake Y., Nakanuma S., Hayashi H., Takamura H., Ninomiya I. (2018). Neoadjuvant chemotherapy with gemcitabine plus nab-paclitaxel reduces the number of cancer-associated fibroblasts through depletion of pancreatic stroma. Anticancer Res..

[37] Torlakovic E.E., Nielsen S., Vyberg M., Taylor C.R. (2015). Getting controls under control: The time is now for immunohistochemistry. J. Clin. Pathol..

[38] Kitano M., Yoshida T., Itonaga M., Tamura T., Hatamaru K., Yamashita Y. (2019). Impact of endoscopic ultrasonography on diagnosis of pancreatic cancer. J. Gastroenterol..

[39] Rodrigues-Pinto E., Grimm I.S., Baron T.H. (2016). Endoscopic ultrasound fine-needle aspiration vs. fine-needle biopsy: Tissue is always the issue. Endosc. Int. Open.

